# Does Resveratrol Impact Oxidative Stress Markers in Patients with Head and Neck Cancer Receiving Home Enteral Nutrition?

**DOI:** 10.3390/nu17030504

**Published:** 2025-01-30

**Authors:** Michał Ławiński, Katarzyna Zadka, Natalia Ksepka, Maima Matin, Kamil Wysocki, Dominika Karkocha, Aleksandra Gradowska, Atanas G. Atanasov, Maciej Słodkowski, Agnieszka Wierzbicka, Artur Jóźwik

**Affiliations:** 1 Department of General, Gastroenterology, and Oncologic Surgery, Medical University of Warsaw, Banacha 1a, 02-097 Warszawa, Poland; michal.lawinski@wum.edu.pl (M.Ł.); dominika.karkocha@gmail.com (D.K.); maciej.slodkowski@wum.edu.pl (M.S.); 2 Department of Biotechnology and Nutrigenomics, Institute of Genetics and Animal Biotechnology, Polish Academy of Sciences, Postępu 36a, Jastrzębiec, 05-552 Magdalenka, Poland; n.ksepka@igbzpan.pl (N.K.); m.matin@igbzpan.pl (M.M.); k.wysocki@igbzpan.pl (K.W.); a.atanasov@igbzpan.pl (A.G.A.); a.wierzbicka@igbzpan.pl (A.W.); aa.jozwik@igbzpan.pl (A.J.); 3 Institute of Applied Psychology, University of Social Sciences, Sienkiewicza 9, 90-113 Łódź, Poland; ola.gradowska@gmail.com; 4 Ludwig Boltzmann Institute Digital Health and Patient Safety, Medical University of Vienna, Spitalgasse 23, 1090 Vienna, Austria; 5 Laboratory of Natural Products and Medicinal Chemistry (LNPMC), Center for Global Health Research, Saveetha Medical College and Hospital, Saveetha Institute of Medical and Technical Sciences (SIMATS), Thandalam, Chennai 602 105, India

**Keywords:** bioactive nutrients, nutraceuticals, resveratrol, oxidative stress, antioxidants, head and neck cancer, home enteral nutrition, malnutrition

## Abstract

**Objectives**: Resveratrol (RES) is well documented for its multiple health benefits, with a notable impact on cancer prevention and therapy. This study aimed to evaluate the effect of RES supplementation on oxidative stress in patients with head and neck cancer (HNC) receiving home enteral nutrition (HEN). **Methods**: This randomized, single-center, open-label study involved 72 adult patients, with 40 completing the intervention. Participants in the intervention group received 400 mg of liposomal RES daily for 12 weeks alongside HEN, while the control group received HEN only. Body composition and oxidative stress markers—including total antioxidant capacity (TAC), malondialdehyde (MDA), superoxide dismutase (SOD), glutathione peroxidase (GPx), and reduced glutathione (GSH)—were measured at baseline and after 12 weeks. **Results**: Significant increases in TAC and SOD activity were observed in both groups. GPx activity increased significantly only in the RES group. MDA levels rose in both groups but were more pronounced in the RES group. GSH levels showed no significant changes. Phase angle (PhA) increased significantly in the RES group, while no significant change was observed in the control group. **Conclusions**: RES supplementation may enhance antioxidant defenses, as evidenced by increased GPx activity and improvements in TAC and SOD levels, supporting oxidative balance in patients with HNC receiving HEN. The higher MDA levels in the RES group may reflect RES’s dual antioxidant and pro-oxidant activities. Additionally, the observed increase in PhA suggests potential cellular health benefits. These findings highlight the potential of RES as a complementary antioxidant intervention in clinical oncology, warranting further investigation to clarify its therapeutic effects on oxidative stress and cellular health in cancer care.

## 1. Introduction

Cancer represents a major global public health issue [1], with head and neck cancers (HNC) accounting for approximately 3% of all cancer cases in 2023 [2,3]. In patients with HNC, malnutrition arises from tumor metabolism, treatment side effects, and swallowing or chewing difficulties [4], affecting 25–65% at diagnosis [5,6,7] and up to 80% as treatment progresses [8]. In patients with HNC, home enteral nutrition (HEN) is crucial for maintaining nutritional status, preventing treatment interruptions, and supporting oxidative balance, thereby helping to stabilize metabolic processes during treatment and recovery [9,10,11]. Common HNC treatments include chemotherapy, radiation, and surgery. Recently, research has highlighted plant-derived compounds, especially polyphenols, for their potential antitumor, anti-inflammatory, immunomodulatory, and antioxidant effects as adjuncts to conventional therapies [12,13,14,15,16,17].

**Figure 1:**
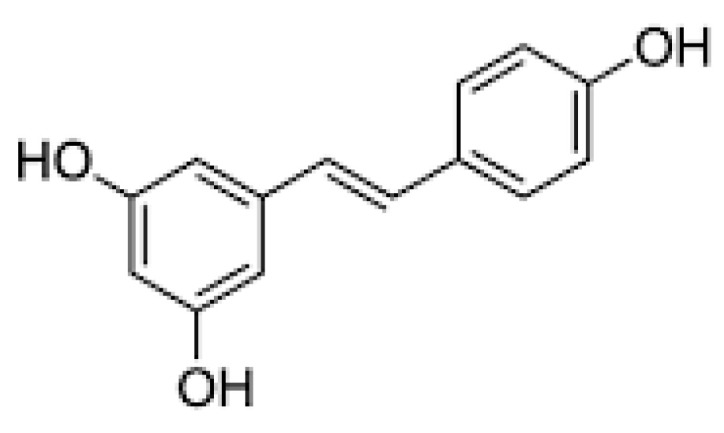
The chemical structures of trans isomer of resveratrol.

One of them seems to be a resveratrol (3,4′,5-trihydroxy-trans-stilbene) (RES), a polyphenolic stilbenoid classified as a phytoalexin (Figure 1). RES was first extracted from white hellebore (*Veratrum grandiflorum*) and Japanese knotweed (*Polygonum cuspidatum*) [18] and is also found in red wine, grapes, berries, tomatoes, and nuts [19]. It is synthesized in response to stress factors like tissue damage and microbial invasion [20].

RES exerts its antioxidant effects [21,22] through various mechanisms, such as preventing lipid peroxidation, enhancing total antioxidant capacity (TAC), and decreasing reactive oxygen species (ROS) production [17,23]. One way is by directly inhibiting the production of ROS via nicotinamide adenine dinucleotide phosphate oxidase, which leads to a decrease in the activity and expression of this enzyme. Additionally, RES reduces the biosynthesis of superoxide and promotes the upregulation of tetrahydrobiopterin synthase as well as guanosine triphosphate cyclohydrolase I while inhibiting endothelial nitric oxide synthase. Furthermore, RES contributes to the upregulation of various antioxidant enzymes and increases the activity of superoxide dismutase (SOD), glutathione peroxidase (GPx), and catalase. This increase in activity subsequently activates a range of nuclear transcription factors, such as Nrf-2, activator protein-1, and SIRT1, which is a crucial metabolic sensor that mediates the response to oxidative stress [24,25,26]. Additionally, RES increase non-enzymatic antioxidants, including reduced glutathione (GSH) [27]. Lastly, studies showed that RES also reduced malondialdehyde (MDA) concentration in serum [28,29].

RES is recognized as a highly effective compound with a broad range of health benefits, including anti-obesity effects and the prevention and management of conditions such as cardiovascular disease, liver disorders, diabetes, neurodegenerative diseases, and cancer [18,20,30,31,32]. RES acts as a chemopreventive agent across all stages of carcinogenesis and has shown efficacy in cancer treatment in both laboratory and animal studies [33].

There is a lack of targeted research examining the specific impact of RES on oxidative stress in patients with HNC receiving HEN. Therefore, the aim of the study was to assess the effect of RES supply on the level of oxidative stress markers in patients with HNC receiving HEN.

## 2. Materials and Methods

### 2.1. Study Design and Ethics Approval

This randomized, interventional, open-label, single-center study was conducted in the Nutrition Clinic of the Department of General, Gastroenterology, and Oncologic Surgery of the UCC Medical University of Warsaw. The study protocol complied with the ethical principles outlined in the Declaration of Helsinki and received approval from the Bioethics Committee at the Medical University of Warsaw (approval number: KB/24/2023; 6 February 2023). Patient enrollment took place over a 12-month period, spanning from February 2023 to February 2024.

### 2.2. Study Population

Patients were included in the study if they met the following criteria: diagnosis of HNC (stage I–IV), age > 18 years, presence of artificial access to the digestive tract, and receipt of partial or total HEN. The exclusion criteria included: diagnosis of a different cancer type, previous cancer diagnosis, life expectancy of less than 3 months, alternative oncological treatments, immunotherapy, ongoing radiotherapy, use of antioxidant-containing supplements, partial or total parenteral nutrition, total oral nutrition, claustrophobia, epilepsy or epileptic episodes, presence of a pacemaker, endoprostheses or devices emitting electrical signals, HBV, HCV, or HIV infection, and pregnancy.

Prior to enrollment, patients received comprehensive information about the study protocol and were required to provide written informed consent. Participants had the right to withdraw from the study at any point without the need to justify their decision.

Patients who agreed to participate in the study were randomly assigned to one of the groups—intervention (RES group) or control group. For this purpose, the block randomization method was used because it allowed for maintaining an equal distribution of patients in the study groups. In order to minimize the predictability of allocation, a variable block size (4 and 6) was used with randomly arranged permutations of possible allocations. Each block contained an equal number of allocations (36 each) to each of the arms (intervention and control).

**Figure 2:**
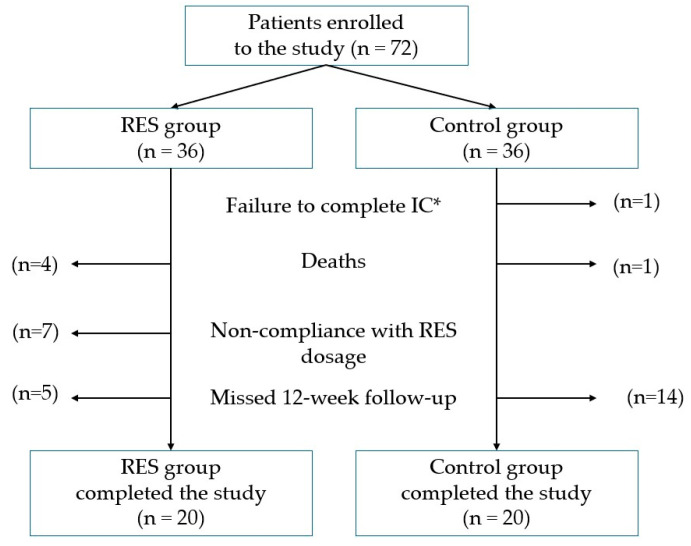
Flow chart showing participant dropout. * IC—indirect calorimetry.

The study included seventy-two adult patients (72.2% male) with a mean age of 64.13 ± 11.40 years diagnosed with HNC who were receiving HEN covered by the National Health Fund. Thirty-two patients did not complete the study. This was due to the following reasons: 1 patient did not complete indirect calorimetry, 7 patients did not comply with the RES dosage recommendations, 5 patients died, and 19 failed to show up for a 12-week follow-up study (Figure 2).

All medical information, including details on disease stage and treatment approach, was obtained from patients’ medical records and interviews. These data were recorded in an anonymized database using a unique code assigned to each patient.

### 2.3. Measurements

All measurements were conducted twice at the beginning of the study and after 12 weeks in the Nutrition Clinic of the Department of General, Gastroenterology, and Oncologic Surgery at the UCC Medical University of Warsaw. At both the beginning and the end of the study, the procedures were performed in the same manner as described below.

#### 2.3.1. Anthropometry

Body weight was assessed using a calibrated electronic scale Tanita WB-380 (Tanita Corporation, Tokyo, Japan), which was adjusted for leveling prior to use. Participants were weighed to the nearest 0.5 kg while barefoot and wearing light clothing. Height was measured using the built-in stadiometer of the same scale, with participants standing in the Frankfurt plane. Height measurements were accurate to 0.5 cm. After both height and weight were recorded, the body mass index (BMI) was calculated and interpreted according to the Centers for Disease Control and Prevention standards [34].

#### 2.3.2. Indirect Calorimetry

To estimate the energy content of enteral nutrition, including HEN, predictive equations are commonly employed. However, these equations are not a flawless alternative to directly measuring energy expenditure. Theoretical predictions lack precision, and the only feasible clinical approach to accurately determine energy expenditure and individualized nutritional therapy is through indirect calorimetry [10]. As the gold standard for assessing resting energy expenditure (REE), indirect calorimetry was utilized in this study. Consequently, all patients included in the personalized nutritional intervention had their REE evaluated using the Q-NRG device (COSMED, Rome, Italy). The measurement followed the guidelines outlined by the ICALIC study group’s position paper [35]. Participants were instructed to avoid strenuous physical activity for 12 h, refrain from eating for at least 5 h, and abstain from caffeine and nicotine for 24 h prior to the test. Before the test began, patients rested quietly in a supine position on a bed for a 15 min acclimation period. The temperature in the examination rooms was controlled between 22 and 25 °C. During the test, patients remained awake and rested in a supine position. The device was calibrated in accordance with the manufacturer’s guidelines 20 min after initialization and prior to performing the measurements. The study used a canopy hood system with a disposable filter and cover, and measurements were collected over 30 min following a 2 min self-calibration of the device. The REE was determined using the abbreviated Weir equation and noted in kilocalories.

#### 2.3.3. Bioelectrical Impedance Analysis

Each patient enrolled in the study to monitor the impact of nutrition on body composition underwent bioelectrical impedance analysis. Body composition was assessed using a phase-sensitive bioimpedance analyzer (AKERN BIA 101, Akern, Pontassieve, Italy), which operates at a single frequency. Patients were instructed to empty their bladder and remain in a supine position for 10 min prior to the procedure to ensure proper fluid balance. Impedance measurements were taken with the patient’s leg positioned at a 45-degree angle from the body’s midline while the arms were placed 30 degrees away from the trunk. The skin was disinfected with alcohol, and two electrodes (Biatrodes, Akern, Pontassieve, Italy) were placed 5 cm apart on the dorsal surface of the right hand and right foot. The assessment generated measurements of fat-free mass (FFM), fat mass (FM), and phase angle (PhA). PhA represents the relationship between resistance and reactance of electrical currents as they traverse body tissues. It is considered a potential indicator of prognosis, health status, functionality, and nutritional condition. A low PhA generally signifies compromised cell membrane integrity, which can impair energy storage and metabolic processes. In contrast, a high PhA reflects well-preserved cell membranes and a greater body cell mass. Since PhA is influenced by tissue composition, including muscle and fat mass, as well as hydration levels, it is hypothesized to serve as a marker of nutritional status [36].

#### 2.3.4. Biochemical Analysis

After anthropometric measurements, indirect calorimetry, and bioelectrical impedance analysis, a blood sample was taken from each patient. Blood samples were collected using S-Monovette^®^ CAT tube syringes (Sarstedt AG & Co. KG, Nümbrecht, Germany) designed for serum analysis to determine C-reactive protein, total protein, albumin, TAC, MDA, and SOD. Blood samples were collected using S-Monovette^®^ K3 EDTA tube syringes (Sarstedt AG & Co. KG, Nümbrecht, Germany) for peripheral whole blood analysis to determine GSH and GPx.

C-reactive protein (CRP), a protein produced by the liver that increases in concentration during inflammatory processes [37], along with total protein and albumin, were measured using standard methods commonly applied in medical testing as part of routine assessments performed within HEN.

TAC, also known as non-enzymatic antioxidant capacity, represents the amount of oxidants neutralized per liter of body fluid. In plasma, non-enzymatic antioxidants consist of both endogenous compounds, such as bilirubin and thiols, and nutritional components, including tocopherols, ascorbic acid, carotenoids, and phenolic compounds [38]. For the determination of TAC, blood samples were centrifuged at 2000× *g* for 15 min at 4 °C. The top yellow serum layer was carefully pipetted without disturbing the white buffy coat. Serum samples were maintained on ice until analysis commenced to prevent any unintended initiation of reactions and diluted with assay buffer before assaying. TAC measurements were performed using the Antioxidant Assay Kit (Item No. 709001) from Cayman Chemical Company (Ann Arbor, MI, USA). Absorbance readings were taken at 750 nm using a Synergy4 microplate reader (BioTek, Winooski, VT, USA). TAC values were derived from a calibration curve created in accordance with the manufacturer’s guidelines and the template included in the assay report. TAC results were expressed in mmol of Trolox equivalent/L.

MDA, a byproduct formed during the peroxidation of polyunsaturated fatty acids in cells, is widely recognized as a marker of oxidative stress and antioxidant status, particularly in cancer patients [39]. To measure the MDA level, before starting the procedure, blood samples were centrifuged at 2000× *g* for 15 min at 4 °C. The top yellow serum layer was carefully pipetted without disturbing the white buffy coat. The supernatant was kept on ice until the start of the analysis. The subsequent procedure followed the manufacturer’s instructions using the TBRAS (TCA Method) Assay Kit (Item No. 700870) from Cayman Chemical Company (Ann Arbor, MI, USA). Absorbance was measured at 535 nm with a Synergy4 microplate reader (BioTek, Winooski, VT, USA). The calculations were derived from a calibration curve developed following the manufacturer’s specifications. The concentration of MDA was expressed in micromoles (µM).

SOD is a metalloenzyme that plays a crucial role in the body’s antioxidant defense against oxidative stress [40]. For the determination of SOD, blood samples were centrifuged at 2000× *g* for 15 min at 4 °C. The top yellow serum layer was carefully pipetted without disturbing the white buffy coat. Serum samples were maintained on ice until analysis commenced to prevent unintended initiation of reactions and were diluted 1:5 with sample buffer before assaying. SOD activity was assessed using the Superoxide Dismutase Assay Kit (Item No. 706002) from Cayman Chemical Company (Ann Arbor, MI, USA). Absorbance readings at 460 nm were obtained using the Synergy4 microplate reader (BioTek, Winooski, VT, USA). The SOD activity was calculated using Gen5 software (BioTek) and expressed in units per milliliter (U/mL).

GPx is an enzyme that prevents the formation of free radicals from hydroperoxides, thereby functioning as an antioxidant [41]. To measure GPx activity, before starting the procedure, the blood samples were centrifuged at 1000× *g* for 10 min at 4 °C. The top yellow plasma layer was carefully pipetted without disturbing the white buffy coat. Samples were diluted 1:2 with Sample Buffer before assaying. The white buffy layer was removed and discarded. The erythrocytes were lysed in 4 volumes of ice-cold HPLC-grade water. The sample was centrifuged at 1000× *g* for 15 min at 4 °C. The supernatant was collected for assaying and stored on ice. The erythrocyte lysate was diluted 1:10–1:20 with Sample Buffer before assaying. The GPx activity was assessed using the Glutathione Peroxidase Assay Kit, Item No. 703102 (Cayman Chemical Company, Ann Arbor, MI, USA). Absorbance was measured at 340 nm with a Synergy4 microplate reader (BioTek, Winooski, VT 05404, USA). GPx levels were calculated based on a calibration curve prepared according to the manufacturer’s instructions. The GPx level was expressed in nmol/min/mL.

GSH plays a vital role in protecting cells from oxidative damage, mitigating the toxicity of xenobiotic electrophiles, and maintaining redox balance [42]. The determination of GSH concentration was performed using the OxisResearch™ Bioxytech^®^ GSH/GSSG—412™ test (Foster City, CA, USA). Whole blood samples were prepared for analysis by adding M2VP (1-methyl-2-vinyl-pyridium trifluoromethanesulfonate) and storing them at −80 °C. The procedure followed the guidelines provided by the kit manufacturer. Absorbance measurements were taken at 412 nm using a Synergy4 microplate reader (BioTek, Winooski, VT, USA). The results were analyzed using the Gen5 software, and the GSH concentration was expressed as thiol groups (µM—SH groups).

### 2.4. Nutritional Intervention

All patients in the RES and control groups were given the same commercial enteral formula in an amount adjusted to the individual energy requirements based on the results of indirect calorimetry. The selected enteral formula was dedicated to oncology patients, nutritionally complete, polymeric, hypercaloric, and high in protein, with added fiber and medium-chain fatty acids. Patients assigned to the intervention group were asked to administer liposomal RES (Kenay^®^ GmbH, Liposomal Resveratrol, Berlin, Germany) into the artificial access to the digestive tract twice daily, 5 mL each, for a total of 10 mL per day. The first dose was administered after the morning intake of the enteral formula, and the second after the last evening intake. The daily dose of RES administered to patients with the supplement was 400 mg. The intervention time for a single patient was 12 weeks.

The decision to use a 400 mg dose reflects both clinical research findings and practical considerations to ensure efficacy and patient safety. Additionally, the 400 mg dose is not only close to the most studied dose of 500 mg, investigated in 32 clinical trials, but also easy for patients to self-administration, as it corresponds to 5 mL of a liquid, commercially available supplement and can be administered through artificial feeding access. Furthermore, recommendations advise against exceeding 1 g per day in healthy populations due to the potential for gastrointestinal side effects and the risk of high doses influencing drug-metabolizing enzyme activity [43]. These considerations are particularly important for patients with HNC, who may be more vulnerable to these effects.

Also, the administration of resveratrol for a period of 12 weeks was based on previous clinical studies that used this duration to evaluate its efficacy and safety [44,45].

The liposomal form of RES provides several advantages over conventional formulations, such as enhanced bioavailability, improved stability, and targeted delivery. For example, liposomal encapsulation has been demonstrated to increase RES bioactivity and stability under UV irradiation. Additionally, it enhances the solubility and absorption of RES, resulting in greater bioavailability and therapeutic efficacy [46,47].

### 2.5. Statistical Analysis

All collected data were summarized using descriptive statistics. Categorical variables are presented as numbers and percentages, while the distribution of numerical data was assessed with the Shapiro–Wilk test. For comparisons between sexes and groups, the *t*-test for independent samples was employed for normally distributed variables, and the Mann–Whitney U test was used for variables without a normal distribution. Within-group differences were evaluated using the *t*-test for dependent samples for normally distributed data and the Wilcoxon signed-rank test for non-normally distributed data. Statistical analyses were conducted using Statistica software (version 13, TIBCO Software Inc., Palo Alto, CA, USA), with a significance level set at 0.05 for all tests.

## 3. Results

### 3.1. Participants Characteristic

A total of 40 patients completed the study—20 receiving RES and 20 from the control group (Figure 2). The study group that completed the research was predominantly male (67.5%), and the mean age was 65.65 ± 11.66 years, and did not differ significantly between the study and control groups. Most patients were at stage III of the disease. The majority of patients were diagnosed with malignant tongue cancer. Treatment predominantly involved surgery followed by radiotherapy or chemoradiotherapy, while in some cases, chemoradiotherapy was the sole approach. The median duration of treatment and HEN showed significant variation between groups, with longer durations observed in the control group. A comprehensive overview of the study population can be found in Table 1.

### 3.2. Anthropometry and Body Composition

At the beginning of the study, there were no statistically significant differences in body weight, BMI, REE, FFM, FM, and PhA between the RES and control groups. After 12 weeks, both groups exhibited increases in body weight and BMI; however, these changes were statistically significant only in the control group. Despite these intra-group differences, no significant differences in body weight or BMI were observed between the groups at the end of the study. REE increased significantly in both groups over the 12-week period. Although REE increased in both the RES and control groups, the proportions of increase were similar, and no significant differences were observed between the groups at the end of the study. In terms of body composition, FFM did not change significantly in either group, although both the RES and control groups showed slight increases. Significant differences were observed in FM and PhA. The control group experienced a significant increase in FM, whereas the RES group showed no significant change. Importantly, PhA increased significantly in the RES group, while the control group showed no significant change (Table 2).

### 3.3. CRP, Total Protein, and Albumin

Biochemical parameters such as CRP, total protein, and albumin did not show significant differences between the RES and control groups at the beginning of the study. After 12 weeks, CRP levels increased in both groups, but this was not statistically significant. Interestingly, total protein levels decreased slightly in the RES group while increasing in the control group, leading to a significant difference between the groups after 12 weeks, though these changes were not statistically significant within the groups. Albumin levels remained stable in both groups throughout the study, with no significant changes within or between the groups (Table 2).

### 3.4. Oxidative Stress

**Figure 3:**
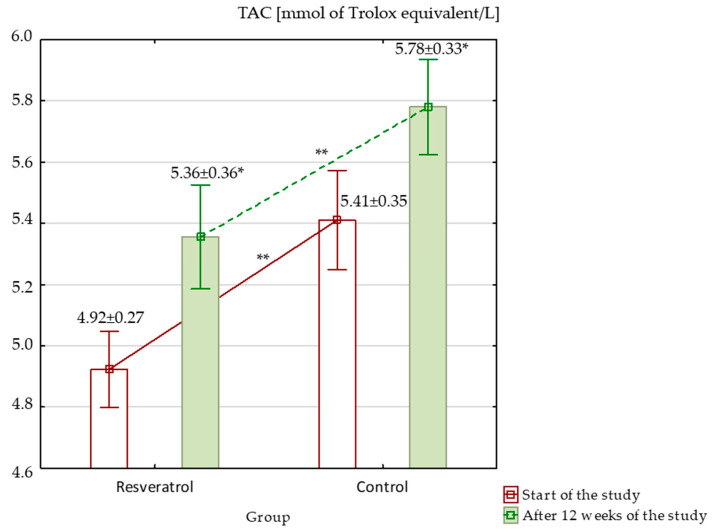
Total antioxidant capacity (TAC) in the resveratrol and control groups measured at baseline and after 12 weeks of intervention. * The statistically significant change within the group: RES group *p* < 0.001; control group *p* = 0.001. ** The statistically significant differences between the groups: start *p* < 0.001; after 12 weeks *p* < 0.001.

**Figure 4:**
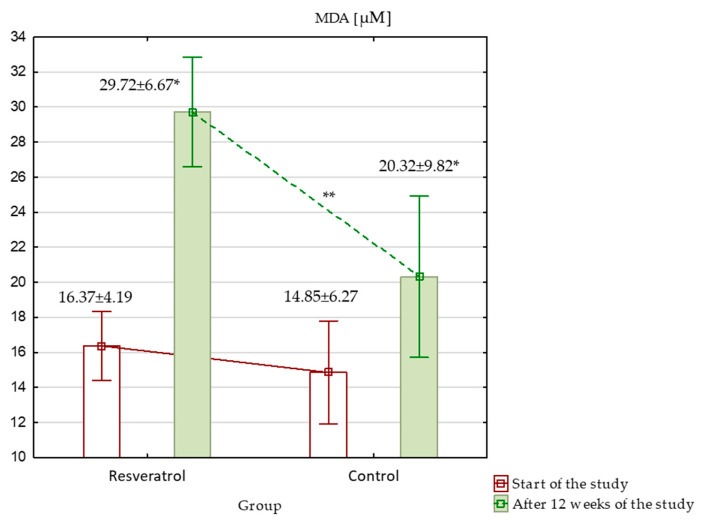
Reduced malondialdehyde (MDA) in the resveratrol and control groups measured at baseline and after 12 weeks of intervention. * The statistically significant change within the group: RES group *p* < 0.001; control group *p* = 0.007. ** The statistically significant differences between the groups: after 12 weeks *p* = 0.001.

**Figure 5:**
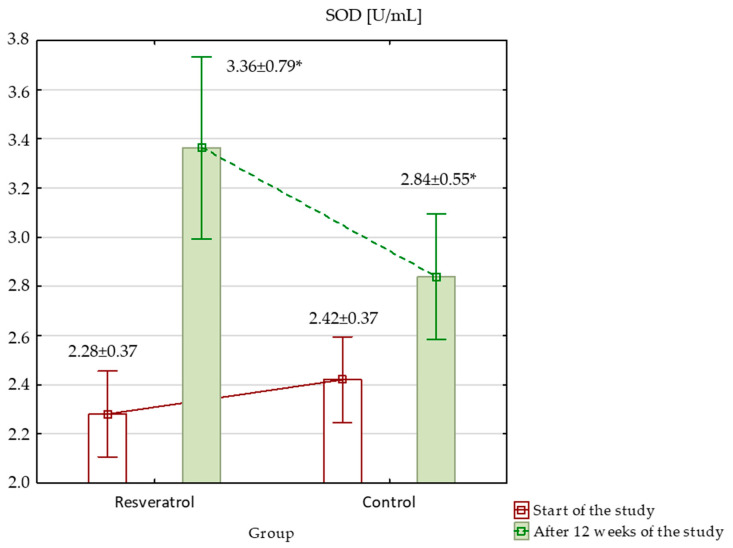
Superoxide dismutase (SOD) in the resveratrol and control groups measured at baseline and after 12 weeks of intervention. * The statistically significant change within the group: RES group *p* < 0.001; control group *p* = 0.001.

**Figure 6:**
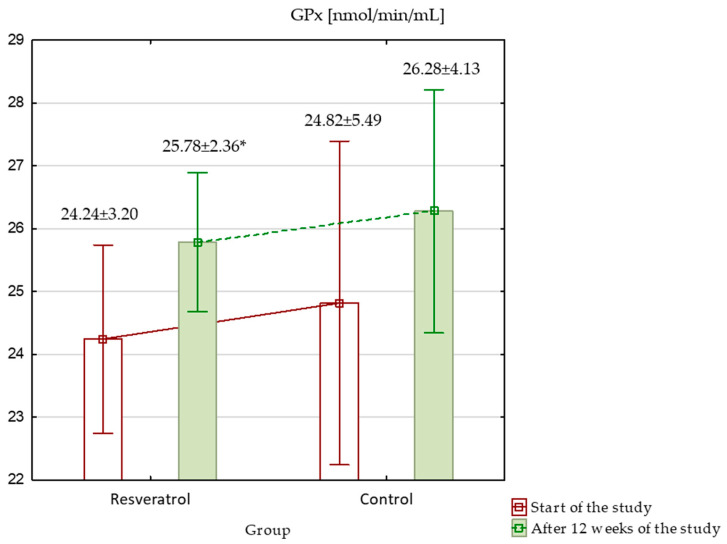
Glutathione peroxidase GPx in the resveratrol and control groups measured at baseline and after 12 weeks of intervention. * The statistically significant change within the group: RES group *p* = 0.02.

**Figure 7:**
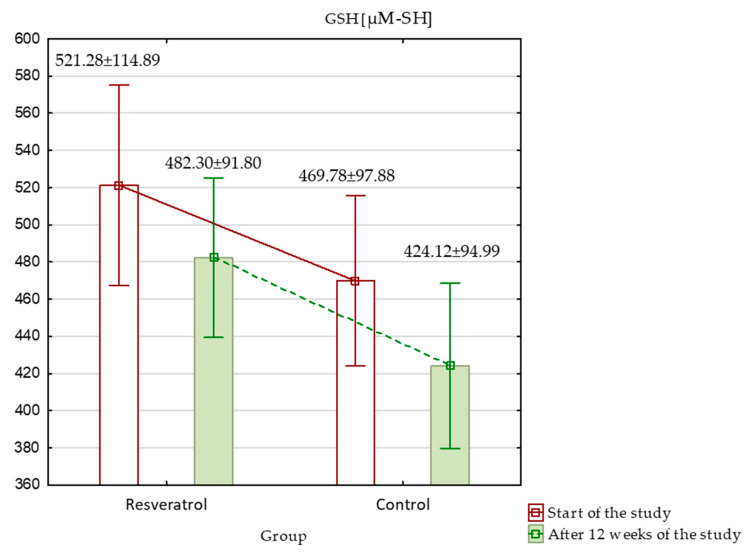
Reduced glutathione (GSH) in the resveratrol and control groups measured at baseline and after 12 weeks of intervention.

When assessing oxidative stress markers at the beginning of the study, TAC was significantly higher in the control group (*p* < 0.001), while other markers, such as MDA, SOD, GPx, and GSH, showed no significant differences between the groups. Over the study period, TAC increased significantly in both groups; however, despite the increase, it remained significantly higher in the control group (Figure 3). MDA level showed no significant differences at baseline. After 12 weeks, MDA levels increased significantly in both groups, with a larger rise in the RES group, resulting in significantly higher MDA levels in the RES group compared to the control at the end of the study (Figure 4). SOD activity was similar between groups at baseline. By week 12, SOD activity increased significantly in both groups. Although these increases occurred, no significant differences in SOD activity were observed between the groups at the end of the study (Figure 5). GPx activity showed no significant differences at baseline. After 12 weeks, GPx activity increased significantly in the RES group, but no significant change was observed in the control group (Figure 6) and between the groups. Lastly, GSH levels were not significantly higher in the RES group at baseline compared to the control group. By the end of the study, GSH levels decreased in both groups. However, these changes were not statistically significant (Figure 7).

In summary, after 12 weeks, the RES group demonstrated significant improvements in several key oxidative stress and body composition markers. PhA increased significantly only in the RES group, while FM remained stable compared to the control group, which experienced an increase. SOD increased in both groups, while GPx rose significantly only in the RES group. TAC was initially higher in the control group at the start of the study and remained higher throughout, even though both groups experienced a significant and similar increase. However, MDA levels, a marker of oxidative damage, worsened more in the RES group compared to the control group. GSH levels decreased in both groups without significant differences.

## 4. Discussion

Our study aimed to evaluate the effects of RES supplementation on various clinical and biochemical parameters in patients with HNC receiving HEN. In the studied population, most participants were men, even though the enrollment spanned one year. This imbalance in sex distribution likely mirrors the disparity in HNC incidence between men and women. Data from the Polish National Cancer Registry, published in 2022, indicate that over 70% of HNC cases recorded in 2020 occurred in men [48]. While the groups were comparable at baseline, notable differences in outcomes began to emerge over the 12-week period, revealing key trends between the RES and control groups.

### 4.1. The Impact of RES on PhA

PhA is recognized as a valuable prognostic tool in HNC, a condition where malnutrition is commonly observed [49]. Moreover, PhA may provide insights into oxidative stress levels [50]. In our study, only the RES group demonstrated an increase in PhA. Considering the complex interconnections between oxidative stress, cellular damage, and inflammation—factors potentially associated with lower PhA values [50]—our results suggest that RES might support oxidative defense by promoting cellular integrity. During oxidative stress, ROS destabilizes cell membranes, disrupting the balance of fluids between intracellular and extracellular spaces. This alteration impacts membrane capacitance, subsequently decreasing PhA [51]. The conducted studies have demonstrated a relationship between PhA and markers of oxidative stress. Zouridakis et al. initially documented a moderate positive correlation between PhA and TAC in patients with chronic kidney disease [52]. Similarly, Tomeleri et al. observed positive correlations between PhA and antioxidants enzymes such as catalase, SOD, and total radical trapping antioxidant in older women with increased BMI and comorbidities [53,54]. To the best of the authors’ knowledge, no studies have investigated the impact of RES supplementation on PhA, particularly in the context of patients with HNC. Only Di Renzo et al. investigated the impact of enteral immunonutrition versus a standard oral diet on PhA and inflammatory response in patients with advanced HNC, demonstrating a positive effect in the studied group [55].

### 4.2. The Impact of RES on FM

In our research into the RES group, FM remained stable, contrasting with the control group, which exhibited a significant increase. It may relate to RES’ potential anti-obesity effects. According to a systematic review, 37 out of 38 in vitro studies reported that RES exerts anti-obesity effects on 3T3-L1 adipocytes through various mechanisms. These mechanisms include the reduction in fat accumulation and adipogenesis, inhibition of preadipocyte proliferation and subsequent differentiation, promotion of white adipocyte browning, induction of apoptosis, and upregulation of miRNAs involved in antiadipogenic pathways and triacylglycerol metabolism within white adipose tissue [56]. Nonetheless, data from human studies remain limited, and findings are often inconsistent and inconclusive [57]. To the authors’ knowledge, no research to date has evaluated the effect of RES supplementation on FM in patients with HNC.

### 4.3. The Impact of RES on TAC, SOD, and GPx

At the start of the study, TAC was notably higher in the control group, while no significant differences were observed for other oxidative stress markers between the groups.

The higher baseline TAC in the control group could have potentially masked the effects of RES in the intervention group, making its impact on oxidative stress markers less pronounced. This discrepancy might have influenced the results for other markers, which could have differed if the baseline TAC had been comparable across groups. In our study, both groups exhibited a significant increase in TAC and SOD levels, while significant elevation in GPx was observed exclusively in the RES group. This outcome may be attributed to the combined beneficial effects of RES and individually tailored HEN on the body’s antioxidant potential in the RES group, and to the impact of HEN alone in the control group. It is known that reducing dietary deficiency not only improves the synthesis of plasma albumin and antioxidant enzymes but also increases tissue concentrations of antioxidants, thereby improving antioxidant status. Plasma albumin, erythrocyte, glutathione, and other endogenous antioxidant molecules directly scavenge ROS [58,59]. At the same time, it was observed that RES notably elevated SOD activity in prostate, liver, and breast cancer cells [12]. Additionally, RES activates the Nrf2/HO-1 signaling pathway, leading to upregulation of SOD and also GPx activity [17]. The authors of a systematic review and meta-analysis of randomized clinical trials also examined the effects of RES on oxidative stress markers. Among six double-blinded randomized clinical trials, they found that RES supplementation significantly increased GPx serum levels (like in our study) but had no significant effect on SOD concentrations, TAC, or MDA serum levels compared to placebo. However, none of these studies involved patients with HNC [60]. The results of another systematic review and meta-analysis evaluating the therapeutic effect of RES supplementation on oxidative stress, this time based on the analysis of twelve randomized clinical trials, showed that RES supplementation significantly increased TAC levels (like in our study), while it did not demonstrate any significant effect on oxidative markers SOD, CAT, and GPx. Similarly, none of the studies analyzed in this case included patients with HNC [17]. To the authors’ knowledge, no research to date has evaluated the effect of RES supplementation on TAC, SOD, and GPx levels in patients with HNC.

### 4.4. The Impact of RES on MDA

MDA is a byproduct of lipid peroxidation involving polyunsaturated fatty acids that contain two or more double bonds separated by methylene groups. This reactive aldehyde can interact with nucleic acids and form covalent adducts with proteins, leading to cellular toxic stress [61]. Regarding MDA, we expected no influence or decrease in the group receiving RES. However, in both groups, MDA levels increased; additionally, this rise was greater in the RES group. The higher increase in MDA levels in the RES group, in our view, may stem from the fact that certain phenolic compounds, including RES, exhibit both antioxidant and pro-oxidant activities. The antioxidant function of phenolic compounds is typically associated with their ability to prevent the oxidation of carbohydrates, proteins, lipids, and DNA, particularly at lower concentrations. In contrast, their pro-oxidant activity is linked to their potential to induce lipid peroxidation, DNA damage, mutagenesis, carcinogenesis, or apoptosis in cancer cells [62]. RES’s impact on mitochondrial oxidative stress seems to specifically harm cancer cells. By intensifying oxidative stress within their mitochondria, RES promotes cellular toxicity, resulting in damage and cancer cell death, often with minimal effects on healthy cells. This compound exhibits a dual action on ROS balance. RES functions as an antioxidant under normal conditions but shifts to a potent pro-oxidant role in cancer cells, activating apoptotic pathways. Furthermore, activation of endogenous antioxidant defense systems through the Nrf2 transcription factor by RES is triggered by the pro-oxidant properties of the phytochemical since exposure to oxidative or electrophilic stress is the primary mechanism activating the Nrf2 nuclear translocation [63]. The anti-cancer effects of RES are thus attributed to both its antioxidant and pro-oxidant properties [64,65,66,67]. To the authors’ knowledge, no research to date has evaluated the effect of RES supplementation on MDA levels in patients with HNC.

### 4.5. The Impact of RES on GSH

GSH levels did not change significantly in either group, suggesting that the effects of RES may be more targeted toward specific oxidative and cellular health parameters rather than broader biochemical markers. Although RES is recognized for its antioxidant properties, there is a lack of specific research examining its impact on GSH levels in human subjects.

In summarizing the study’s findings, several limitations should be considered. The sample size was limited, with only 40 patients completing the study due to non-compliance, mortality, and loss of follow-up, which may reduce the statistical power and limit the generalizability of the results. Additionally, this research focused exclusively on patients with HNC receiving HEN, restricting the applicability of findings to other cancer types or patients with different nutritional needs. Furthermore, the intervention duration of 12 weeks may not capture the potential long-term effects of RES on oxidative stress markers, body composition, or broader health outcomes.

## 5. Conclusions

This study suggests that RES supplementation, administered alongside HEN in patients with HNC, may support improvements in specific oxidative stress markers and body composition. Increases in both TAC and SOD were observed in both groups, suggesting that HEN alone has a positive impact on antioxidant capacity and enzymatic defense against oxidative stress in patients with HNC. However, a significant rise in GPx activity was observed exclusively in the RES group. This selective effect on GPx suggests that RES could offer additional, targeted antioxidant benefits beyond the standard nutritional support provided by HEN. The higher increase in MDA levels in the RES group may stem from the fact that certain phenolic compounds, including RES, exhibit both antioxidant and pro-oxidant activities. Additionally, the exclusive increase in PhA in the RES group points to a potential role of RES in enhancing cellular resilience and function.

These findings highlight RES’s possible utility as a complementary agent in nutritional interventions for oncology patients. Given the limited scope of existing research, further studies are needed to better understand the therapeutic value of RES in managing oxidative stress and supporting patient outcomes in clinical oncology.

## Figures and Tables

**Table 1:** Detailed description of the study population that completed the study (*n* = 40).

Variable		Total (n= 40)			RES Group (n = 20)			Control Group (n = 20)			*t*-Test or U Mann–Whitney Test *
		n	%	Mdn (IQR)/M ± SD *	n	%	Mdn (IQR)/M ± SD *	n	%	Mdn (IQR)/M ± SD *	
Sex	Female	13	32.5	-	6	30.0	-	7	35.0	-	-
	Male	27	67.5		14	70.0		13	65.0		
Age [years]	30–50	5	12.5	65.65 ± 11.66	2	10.0	66.95 ± 10.36	3	15.0	64.35 ± 12.97	*p* = 0.49
	51–70	19	47.5		11	55.0		8	40.0		
	71–90	16	40.0		7	35.0		9	45.0		
Site of primary tumor	Tongue	11	27.5	-	4	20.0	-	7	35.0	-	-
	Throat	8	20.0		5	25.0		3	15.0		
	Tonsil	7	17.5		3	15.0		4	20.0		
	Larynx	5	12.5		3	15.0		2	10.0		
	Oral cavity	5	12.5		4	20.0		1	5.0		
	Other	4	10.0		1	5.0		3	15.0		
Cancer stage	I	1	2.5	-	0	0.0	-	1	5.0	-	-
	II	14	35.0		7	35.0		7	35.0		
	III	17	42.5		9	45.0		8	40.0		
	IV	8	20.0		4	20.0		4	20.0		
Mode of treatment	Surgery (S)	4	10.0	-	1	5.0	-	3	15.0	-	-
	Chemotherapy (Ch)	2	5.0		0	0.0		2	10.0		
	Radiotherapy (R)	4	10.0		3	15.0		1	5.0		
	Chemoradiotherapy (ChR)	7	17.5		5	25.0		2	10.0		
	S + R	8	20.0		4	20.0		4	20.0		
	S + ChR	7	17.5		4	20.0		3	15.0		
	Ch + R	2	5.0		0	0.0		2	10.0		
	Ch + ChR	4	10.0		3	15.0		1	5.0		
	More than 2 methods	2	5.0		0	0.0		2	10.0		
Duration of disease [days]	Less than 3 months	1	2.5	272.5 (155.5–668.0)	0	0.0	416 (220.0–1153.0)	1	5.0	199.5 (126.0–326.5)	*p* = 0.006
	3–6 months	12	30.0		5	25.0		7	35.0		
	7–12 months	11	27.5		4	20.0		7	35.0		
	13–24 months	7	17.5		4	20.0		3	15.0		
	More than 24 months	9	22.5		7	35.0		2	10.0		
Duration of HEN [days]	Less than 3 months	11	27.5	157 (80.5–417.0)	2	10.0	339.0 (132.5–1067.5)	9	45.0	92.0 (49.5–161.5)	*p* < 0.001
	3–6 months	12	30.0		5	25.0		7	35.0		
	7–12 months	6	15.0		4	20.0		2	10.0		
	13–24 months	4	10.0		3	15.0		1	5.0		
	More than 24 months	7	17.5		6	30.0		1	5.0		

HEN—home enteral nutrition. RES—resveratrol. Mdn—median. M—mean. SD—standard deviation. IQR—the interquartile range. * Depending on the distribution of the variable.

**Table 2:** Anthropometric and biochemic parameters of the study population that completed the study (*n* = 40).

	Start of the Study			After 12 Weeks of the Study			Significance of Changes Within the Groups over 12 Weeks ***	
	RES Group (*n* = 20) Mdn (IQR)/M ± SD *	Control Group (*n* = 20) Mdn (IQR)/M ± SD *	Significance of Differences Between the Groups **	RES Group (*n* = 20) Mdn (IQR)/M ± SD *	Control Group (*n* = 20) Mdn (IQR)/M ± SD *	Significance of Differences Between the Groups **	RES Group (*n* = 20)	Control Group (*n* = 20)
Body weight [kg]	63.72 ± 11.30	63.21 ± 13.92	*p* = 0.35	64.40 ± 11.54	65.56 ± 15.20	*p* = 0.79	*p* = 0.35	*p* = 0.003
BMI [kg/m^2^]	22.93 ± 2.79	22.50 ± 3.34	*p* = 0.66	23.17 ± 2.92	23.28 ± 3.57	*p* = 0.92	*p* = 0.35	*p* = 0.004
REE [kcal/day]	1461 ± 336	1502 ± 481	*p* = 0.76	1581 ± 309	1686 ± 582	*p* = 0.48	*p* = 0.02	*p* = 0.03
FFM [kg]	45.68 ± 8.80	47.20 ± 13.03	*p* = 0.67	46.10 ± 8.30	47.31 ± 13.46	*p* = 0.73	*p* = 0.41	*p* = 0.87
FM [kg]	18.04 ± 4.89	16.02 ± 5.01	*p* = 0.20	18.30 ± 5.46	18.26 ± 5.43	*p* = 0.98	*p* = 0.69	*p* = 0.001
PhA [°]	5.12 ± 0.73	5.13 ± 0.88	*p* = 0.98	5.77 ± 0.89	5.39 ± 0.89	*p* = 0.18	*p* = 0.004	*p* = 0.11
CRP [mg/L]	2.50 (1.00–7.85)	3.15 (1.00–26.65)	*p* = 0.29	3.95 (1.65–12.10)	4.10 (1.15–17.65)	*p* = 0.89	*p* = 0.09	*p* = 0.42
Total protein [g/dL]	7.24 ± 0.36	7.28 ± 0.55	*p* = 0.79	7.12 ± 0.27	7.44 ± 0.53	*p* = 0.02	*p* = 0.13	*p* = 0.29
Albumin [g/dL]	4.55 (4.35–4.70)	4.50 (4.35–4.70)	*p* = 0.88	4.55 (4.40–4.80)	4.50 (4.40–4.70)	*p* = 0.46	*p* = 0.67	*p* = 0.70

BMI—body mass index: underweight (BMI < 18.5 kg/m^2^), normal (BMI 18.5 kg/m^2^–24.9 kg/m^2^), overweight (BMI 24.9 kg/m^2^–29.9 kg/m^2^), obesity (BMI > 30 kg/m^2^). REE—resting energy expenditure [kcal/day]. FFM—fat-free mass [kg]. FM—fat mass [kg]. PhA—phase angle [°]. CRP—C-reactive protein. RES—resveratrol. Mdn—median. M—mean. SD—standard deviation. IQR—the interquartile range. * Depending on the distribution of the variable. ** *t*-test or Wilcoxon. *** *t*-test or U Mann–Whitney.

## Data Availability

Data will be available upon reasonable request to the corresponding author.

## Abbreviations

The following abbreviations are used in this manuscript:

BMI	body mass index
FFM	fat-free mass
FM	fat mass
GPx	glutathione peroxidase
GSH	reduced glutathione
HEN	home enteral nutrition
HNC	head and neck cancers
MDA	malondialdehyde
PhA	phase angle
REE	resting energy expenditure
RES	resveratrol
ROS	reactive oxygen species
SOD	superoxide dismutase
TAC	total antioxidant capacity
